# Giant cell tumor of the clivus: A case report and review of the literature

**DOI:** 10.3892/ol.2014.2528

**Published:** 2014-09-12

**Authors:** JING ZHAO, TAO QIAN, ZHENG ZHI, QINGXIA LI, LIN KANG, JUAN WANG, AIXIA SUI, NA LI, HONGTAO ZHANG

**Affiliations:** 1 The First Department of Oncology, Hebei General Hospital, Shijiazhuang, Hebei 050051, P.R. China; 2 Department of Neurosurgery, Hebei General Hospital, Shijiazhuang, Hebei 050051, P.R. China; 3 Department of History and Literature, Hebei University of Traditional Chinese Medicine, Shijiazhuang, Hebei 050200, P.R. China; 4 Department of Pathology, Hebei General Hospital, Shijiazhuang, Hebei 050051, P.R. China

**Keywords:** giant cell tumor, clivus, trigeminal nerve, abducens nerve, radiotherapy, bisphosphonate

## Abstract

Giant cell tumors (GCTs) mainly occur in metaphyses of long bones and are generally considered histologically benign; however, GCTs may be locally aggressive with a high rate of local recurrence and exhibit the potential for distant metastasis. Primary GCT of the clivus is extremely rare and is easily misdiagnosed and, thus, treatment remains controversial. The present report describes the case of a 22-year-old male with GCT located in the skull base originating from the clivus, with the involvement of multiple cranial nerves, which was successfully treated with transnasal transsphenoidal surgery following adjuvant radiotherapy and intravenous bisphosphonate administration. The patient remains symptom free at two years of follow-up. This report contributes to the limited literature regarding GCTs of the skull.

## Introduction

Giant cell tumors (GCTs) are considered to be a locally aggressive benign tumors, also known as osteoclastoma, which typically occur in the epiphyses of long bones, particularly the distal femur, proximal tibia, distal radius and proximal humerus. GCT rarely manifests in the skull, accounting for <1% of all GCTs of the bone, primarily involving the sphenoid and temporal bones in the middle of the cranial fossa (1–3). At present, the majority of studies regarding GCT of the skull are case reports, and the bones involved include temporal bone, petrosal bone, sphenoid and occipital bone. Primary GCT of the clivus is extremely rare. Due to the small number of skull GCTs reported in the literature, standard treatments remain unclear, and the efficacy of surgery as well as adjuvant therapies remains undefined. The current study presents a case of GCT in the clivus presenting with abducens nerve and trigeminal nerve involvement concurrently in a 22-year-old male, who was treated successfully with minimally invasive surgery, adjuvant radiotherapy and intravenous bisphosphonates, and the literature regarding diagnosis, treatment and prognosis has been reviewed. Written informed consent was obtained from the patient’s family.

## Case report

**Figure 1:**
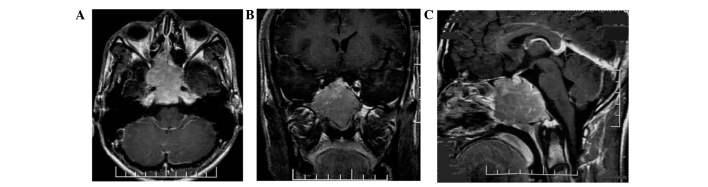
(A) Axial, (B) coronal and (C) sagittal section of T1-weighted magnetic resonance imaging with contrast revealing an expansive tumor originating from the clivus and surrounding both cavernous sinuses, compressing the front of optic chiasm in the sphenoid sinus area of the middle fossa.

A 22-year-old male with a six month history of dull frontal headache did not receive any medical treatment or examination as the symptoms were tolerable. However, three days prior to admission, the headache worsened, and was localized to the right side. The patient developed secondary diplopia and facial numbness in the right maxilla area. On ophthalmological examination, the diplopia secondary to the left abducens nerve (CN VI) palsy was observed in the left eye. Examination of the cranial nerves revealed facial paresthesia along the distribution of maxillary (V2) divisions of the right trigeminal nerve (CN V). No abnormalities in vision, visual field, corneal reflexes, hearing or the power of masseters were identified and no papilledema was observed. The remaining motor and sensory neurological examinations, including cerebellar tests, were normal with full cooperation and orientation. Endoscopic nasal examination revealed a soft, friable mass, which bled when palpated in the posterior wall of the nasopharynx top. The laboratory evaluations including, complete blood count, biochemistry, analysis of tumor markers, thyroid and pituitary function tests and endocrinology examinations were normal, and the patient’s medical history was noncontributory. Magnetic resonance imaging (MRI) of the brain demonstrated an extensive soft-tissue density mass with an irregular shape and a clear boundary measuring 4.0×4.68×3.7 cm, involving the clivus, surrounding the cavernous sinuses on both sides and compressing the front of optic chiasm in the sphenoid sinus area of middle fossa. The posterior wall of the nasopharynx top was not involved. The tumor tissue was isointense on T1-weighted imaging (WI), T2WI and fluid-attenuated inversion recovery, and moderate homogenous enhancement was identified on the post contrast scan (Fig. 1). Due to the symptoms of the present illness and MRI imaging, chordoma and malignant tumor in clivus could not be excluded.

The tumor was removed using an endoscopic transnasal transsphenoidal method under general anesthesia. Intraoperative findings revealed a gray, soft, friable, hypervascular mass arising from the clivus and involving the sphenoid and posterior ethmoid sinuses. Consequently, almost total resection included the tumor together with the slopes and bone surrounding the optic canal and all cranial nerves were preserved. Postoperatively, the patient exhibited transient diabetes insipidus.

From the postoperative histopathology, the patient was diagnosed with a giant cell tumor. The histopathology revealed the tumor was composed of flaky oval or spindle-shaped mononuclear stromal cells and evenly distributed osteoclast-like multinucleated giant cells. The giant cells contained a variable number of nuclei with an median of 25 (range, 10–30). The nuclear features of stromal cells were similar to multinucleated giant cells, with open chromatin, one to two nucleoli and numerous mitotic figures (≤15 per 10 high-power fields). No pathological mitosis was identified (Fig. 3).

Postoperatively, the patient was administered three courses of intravenous zoledronate (4 mg, once a month) and radiotherapy to reduce the local recurrence caused by subtotal resection of the tumor. The patient received three-dimensional conformal radiotherapy using five coplanar fields and two non-coplanar fields to deliver a total dose of 45 Gy in 25 fractions over five weeks. The dose volume histogram revealed that 92% of the planning treatment volume was receiving 100% of the dose. All critical structures received doses within their limits of tolerance. The patient completed therapy without any significant acute toxicity. Complete regression of the tumor was later confirmed on MRI following treatment.

**Figure 2:**
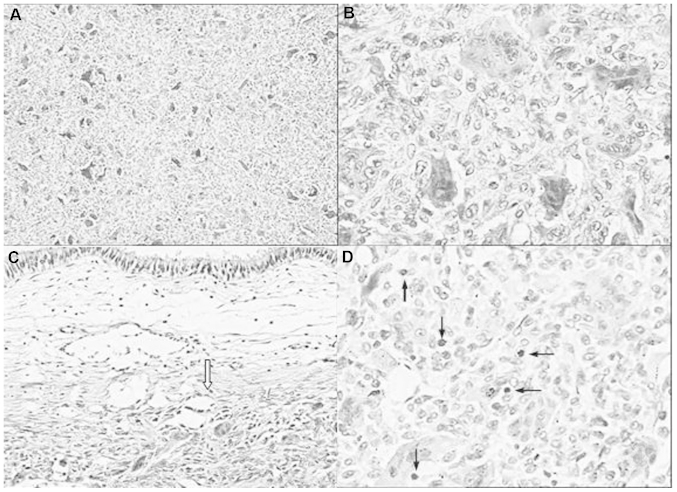
(A and B) Histopathological examination revealed that the tumor was composed of multinucleated giant cells and proliferative oval or spindle-shaped mononuclear stromal cells (stain, hematoxylin and eosin; magnification, A, ×40; B, ×200). (C) The tumor was locally aggressive infiltrating submucosal glands (stain, hematoxylin and eosin; magnification, ×100). (D) Numerous mitotic figures (arrows) in mononuclear stromal cells were observed; up to five per high-power field (stain, hematoxylin and eosin; magnification,x200).

**Figure 3:**
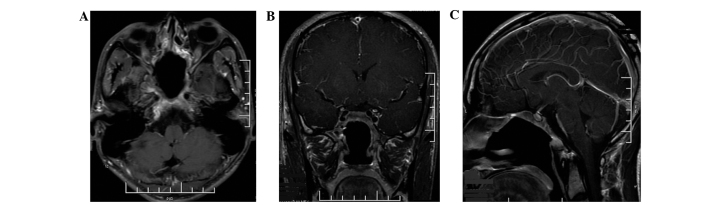
(A) Axial, (B) coronal and (C) sagittal section of postoperative T1-weighted magnetic resonance imaging with contrast revealing no residual or recurrent tumor.

Follow-up has been performed for two years, the patient is clinically asymptomatic and no evidence of recurrence or metastases has been identified by computed tomography (CT) and MRI examination (Fig. 2).

## Discussion

GCTs are generally considered histologically benign; however, they may exhibits locally aggressive behavior with a high rate of local recurrence of up to 60% if treated purely by intralesional curettage. In adiditon, GCTs exhibit the potential for distant metastasis, mostly commonly to the lung, which occurs in 4% of patients with GCT (7). The incidence of GCT is low, accounting for only ~4–5% of primary tumors of the skeleton; however, it is relatively more common in Asian populations, accounting for 14.2%of primary tumors of the skeleton in China, and it occurs more frequently in females than in males, between the second and fourth decades of life following skeletal maturation (8). GCTs most frequently occur in the metaphyses of long bones, but rarely in the skull, accounting for <1% of bone GCTs, where it is usually located in sphenoid and temporal bone. However, in the present case, the tumor primarily arose from the clivus with sellar extension. During the past decade, only five cases of primary clival GCT have been reported (Table I).

Clinical manifestations are usually in accordance with the site of the tumor. Skull-base GCTs generally present with headache, decreased vision, visual field defect, diplopia, ophthalmoplegia, deafness, endocrinopathy and dysfunction of cranial nerves, most commonly the sixth followed by the third cranial nerve (6). However, our patient developed diplopia and facial numbness, which indicated the involvement of the sixth and the fifth cranial nerves. Similar cases with sixth and fifth cranial nerve involvement concurrently have rarely been reported (3,9).

X-ray and CT scan of skull GCTs frequently demonstrate an expansive and occasional lytic bone lesions usually without the classical ‘soap bubble’ appearance. MRI clearly demonstrates the soft-tissue extension and association with the surrounding structures. On MRI, GCTs are usually hypointense or isointense on T1-weighted images (WI) and T2WI with contrast enhancement (10,11). A similar pattern was observed in the present case. The major radiological differential diagnoses include chordoma, giant-cell reparative granuloma, aneurysmal bone cyst, fibrous dysplasia, ‘brown tumor’ of hyperparathyroidism, eosinophilic granuloma and plasmacytoma. Imaging examination alone is insufficient to differentiate these lesions and, thus, the final diagnosis is dependent on histopathology.

GCTs of the bone originate from the primary mesenchymal stromal cells in the connective tissue of the bone marrow, which expresses the receptor activator of NF-κB ligand that stimulates osteoclast maturation from mononuclear precursors. Histologically, GCTs are primarily composed of mononuclear stromal cells and giant cells. However, histogenesis is controversial as the terms GCT and osetoclastoma imply that the giant cells are responsible for the proliferative capacity of the tumor, however, the mononuclear cells present the true neoplastic component and the multinucleated giant cells exhibit an osetoclast-like phenotype and express histocytic lineage markers. The mononuclear cell presents the true neoplastic component while the multinucleated giant cells exhibit an osteoclast-like phenotype and express histocytic lineage markers (12). Multiple cytogenetic abnormalities associated with GCTs have been reported, in which telomere adhesion was the most frequent chromosomal aberration (75%) (13). Cellular morphology is sufficient for the diagnosis of GCT, and immunochemistry is not essential. However, the lineage of these cells may be determined using histiocytic marker CD68 immunostain (12).

The clinical behavior of GCT is unpredictable and, thus, treatment remains controversial. Radical surgical extirpation is the treatment of choice for cranial GCT, which requires complete removal of the diseased bone. However, this may not be possible due to anatomical location or the involvement of vital structures, as observed in the present case, and thus the patient was treated using a minimally intralesional approach. Therefore, the recurrence rate is very high, and the use of adjuvant therapy is invaluable (14,15).

GCTs were previously considered to be radio-resistant with a potential for sarcomatous transformation following radiotherapy. However, along with the development of modern megavoltage irradiation and precise image guided system, the tumor control rate has significantly improved and the frequency of malignant transformation has reduced. Therefore, radiotherapy is recommended as a postoperative adjunctive therapy particularly for incomplete resection in skull base, with a dose of 45–50 Gy in order to gain a long-term healing (16). The role of chemotherapy remains unclear and controversial. A small number of studies have demonstrated that chemotherapy results in good control of primary or recurrent GCT of the skull base (17–19). At present no effective chemotherapeutic agents have been identified for the treatment of this tumor. We suggest that systemic chemotherapy be considered if local control fails following radiotherapy or distal metastases are identified. Studies have indicated that topical or systemic use of bisphosphonates may present a novel adjuvant therapy for GCT by inducing apoptosis of stromal tumor cells and stimulating osteogenic differentiation of the remaining tumor stromal cells following surgery (20–22).

Radiographical and histological grading systems do not predict clinical outcome; however, the extent of surgical resection has been shown to affect prognosis. The majority of recurrences occur within the first two years following treatment, although late recurrences have also been reported and, thus, long-term surveillance is recommended (23).

Giant cell tumors are generally benign, locally aggressive lesions with a potential to metastasize. Primary GCTs of the clivus are extremely rare and only five cases have been reported during the past decade. Imaging examination alone is insufficient for the diagnosis of GCT in the skull-base, and the final diagnosis is dependent on histopathology. Surgical extirpation is the standard treatment for skull-base GCT, and adjuvant radiation must be applied in all cases due to the high rate of local recurrence and since complete resection cannot be achieved. Bisphosphonate administration is also recommended. The present case indicated that the use of subtotal excision via minimally invasive surgery following the administration of intravenous bisphosphonate and adjuvant radiotherapy (45 Gy) results in excellent tumor control after a period of two years’ follow-up. Recurrences usually occur within the first two years following treatment, however, long-term surveillance is proposed.

## Figures and Tables

**Table I:** Summary of giant cell tumor of clivus reported in the English literature.

No.	Author (ref.)	Patient age (years); Gender	Location	Presentations	Therapy	Histology	Recurrence	Patient status; Follow-up
1	Zorlu *et al* (4)	14; Female	Sphenoid, clivus	Headache, diplopia	Neuro-navigation guided transsphenoidal Surgery and radiotherapy (60 Gy) on recurrence	Malignant GCT	Recurrent both after surgery and after radiotherapy	AWD; 2 years
2	Gupta *et al* (12)	17; Female	Clivus	Diplopia, amenorrhea, decreased vision, headache (bilateral CN6 palsy and left CN5 palsy partially)	Surgery via LeFort I osteotomyand and radiotherapy (45 Gy)	Malignant GCT	No	ANED; 2 years
3	Sasagawa *et al* (5)	26; Male	Clivus	Headache, diplopia (right CN6 palsy)	Transsphenoidal surgery and radiotherapy (50 Gy); Transsphenoidal surgery, chemotherapy and artery embolization after recurrence	GCT, osteosarcoma after recurrence	Malignant transformation and lung metastasis	DWD; 10 years
4	Roy *et al* (3)	19; Male	Clivus	Headache, forehead and cheek numbness (right CN5 palsy)	Surgery via right trans-maxillary approach and radiotherapy (45 Gy)	GCT	No	ANED; 18 months
5	Iacoangeli *et al* (6)	31; Male	Clivus	Headache, diplopia (right CN6 palsy)	Surgery via endoscopic extended endonasal approach (EEA)	GCT	No	ANED; 6 years

GCT, giant cell tumor; AWD, alive with disease; ANED, alive with no evidence of disease; DWD, dead with disease.
